# Modified Cellulose-Based Waste for Enhanced Adsorption of Selected Heavy Metals from Wastewater

**DOI:** 10.3390/polym16182610

**Published:** 2024-09-14

**Authors:** Katarina Trivunac, Snežana Mihajlović, Marija Vukčević, Marina Maletić, Biljana Pejić, Ana Kalijadis, Aleksandra Perić Grujić

**Affiliations:** 1 Faculty of Technology and Metallurgy, University of Belgrade, Karnegijeva 4, 11000 Belgrade, Serbiamarijab@tmf.bg.ac.rs (M.V.); biljanap@tmf.bg.ac.rs (B.P.); alexp@tmf.bg.ac.rs (A.P.G.); 2 Innovation Center, Faculty of Technology and Metallurgy, Karnegijeva 4, 11000 Belgrade, Serbia; mvukasinovic@tmf.bg.ac.rs; 3 Textile School for Design, Technology and Management, Academy of Technical and Art Applied Studies Belgrade, StarineNovaka 24, 11000 Belgrade, Serbia; 4 Department of Materials, “VINCA” Institute of Nuclear Sciences—National Institute of the Republic of Serbia, University of Belgrade, Mike Petrovica Alasa 12-14, 11000 Belgrade, Serbia; ana.kalijadis@vin.bg.ac.rs

**Keywords:** waste, polysaccharide-based material, cotton, hydrothermal carbonization, heavy metal, adsorption, wastewater

## Abstract

Due to industrial growth and its impact on the environment, the increasing amount of industrial waste requires a comprehensive approach aligned with the principles of sustainable development. The main goals are not only to preserve natural resources but also to encourage innovation in the reuse of waste materials. In an attempt to reduce the problems regarding waste disposal and wastewater treatment in the textile industry, fibrous textile waste was used as a starting material to obtain carbon adsorbents for the removal of pollutants from wastewater. Waste cotton and mixed yarns, mainly consisting of polysaccharide cellulose, were hydrothermally carbonized and activated with KOH to convert them into efficient carbon adsorbents for heavy metal removal from water. Characterization of carbonized material showed that after activation, an increase in specific surface area (up to 872 m^2^/g) and content of surface oxygen groups (6.04 mmol/g) leads to a higher affinity towards heavy metal ions, especially lead ions, and high adsorption capacity of 19.98 mg/g obtained for activated cotton yarns. The results of this research represent a contribution to the reduction of waste materials by modifying them into adsorbents, while the regeneration of adsorbents is an example of the practical application of polysaccharide-based materials in the purification of wastewater containing various heavy metal ions.

## 1. Introduction

During different stages of industrial production (textile, paper, decorative fabrics), various cellulose-based waste materials, in the form of fibers, yarns, and woven, are generated and disposed of, endangering human health or harming the environment. Furthermore, the textile industry generates large amounts of wastewater, polluted with various hazardous and harmful substances [1,2]. The long-term goals of the European Union’s waste policy are not only to reduce the amount of waste generated but to promote the use of waste as a potential resource, as well as to achieve a higher level of recycling and safe disposal [3]. Waste policy aims to protect the environment and human health while facilitating the transition to a circular economy. Cellulosic materials represent the most widespread natural, renewable, biodegradable, and low-cost raw material. The main component of these materials is the polysaccharide cellulose, a linear homopolymer consisting of β-D-anhydroglucopyranose repeating units linked by β-1,4-glycosidic bonds [4]. It can be obtained from various sources: higher plants, trees, algae, bacteria, and even some marine animals. Cotton fibers are mainly made of cellulose. Polysaccharide-based materials are widespread and widely used (biomedicine, electronics and printing, food, for the production of packaging, biofuels, sorbents, etc.) [5,6] and, therefore, accumulate in large quantities in the form of residues, unused and waste materials. Considering the forecasts that the global production and consumption of fibers will increase until 2030, an increase in the amount of waste cotton and polyester fibers and their blends must be expected [7]. Textile waste is recognized as a significant polluter of the environment due to the large quantities generated during production and after use. Today’s textile and fashion industry implies mass and cheap production using inferior materials, chemicals, and dyes. That is why, in addition to natural materials such as cotton, linen, and hemp, artificial, synthetic materials are also used, which are mixed with natural ones to improve certain properties such as strength, color fastness, and behavior during washing and drying. Polyester fibers are the most popular synthetic textile material due to their physical properties, low cost, and recyclability, but they are not biodegradable, so they stay longer in the environment. Considering that the majority of waste cotton and polyester is mostly burned and disposed of in landfills, it is very important to implement their more massive recycling and reuse in order to reduce negative impacts on water, soil, air, survival of the living world, and human health [8,9,10,11]. Materials produced from biomass or industrial waste can be used as fuels [12,13], catalysts [14], flame retardancy [15], composites, raw material for the production of 5′-hydroxymethylfurfural [16], electrocatalytic carbon material [13], etc. Also, its use in the biomedical field and environmental treatment is significant [14,17,18,19,20]. One possibility is to use these waste materials as adsorbents for purifying water contaminated with different pollutants [8,21]. In the case of raw waste cellulosic materials, the adsorption processes take place mostly slowly, and they have low selectivity and relatively low adsorption capacities. Therefore, for the application of these materials, it is necessary to perform chemical or thermal modifications to obtain efficient, easily applicable materials with increased affinity for pollutant binding thanks to their specific chemical structure, i.e., the presence of different functional groups. Cotton stalk, cotton fibers, denim, household and industrial textile waste, and cotton cloth can be used as raw materials for the production of cotton-based adsorbents. Niu et al. prepared functional cotton fiber with tetraethylenepentamine and chitosan and investigated their adsorption capacity towards Cu(II), Pb(II), and Cr(III) ions. The maximum adsorption capacity obtained by the Langmuir model was highest for Cu(II) ions. Chemical modification of waste textile fibers was performed by the carboxymethylation process [22]. Obtained materials were used for the adsorption of Cd(II) ions and showed great potential for the removal of heavy metals in comparison to commercial resin. The graft copolymerization of household and industrial textile waste by polyacrylic acid was used to prepare adsorbents for the removal of Pb(II) and Cr(VI) ions from water [23]. The maximum adsorption capacity for Pb(II) ions was ten times higher. Modified cotton-based materials showed an increase in affinity towards pollutants than the initial, unmodified ones. Chemical modification has advantages due to a large number of potential reagents and the possibility of obtaining selective adsorbents, but a major disadvantage is the creation of new waste after the adsorption process and secondary pollution. Also, the procurement and storage of chemicals are not economically profitable. The high carbon content in the composition of waste biomass makes them a good starting material for obtaining carbon adsorbents with a large specific surface area and porous structure [24]. The thermal modification of cotton is most often performed using carbonization with pyrolysis and hydrothermal treatment. Pyrolysis of cotton stalks was performed in the temperature range of 250 to 650 °C to obtain biochar, and it was found that materials obtained at temperatures higher than 450 °C had better adsorption characteristics [25]. Cotton waste was transformed into a carbon microtube by direct pyrolysis at 900, 1100, 1300, and 1500 °C and used for sorption of tannic acid [14]. Recently, research has been directed at hydrothermal modification of cotton from different sources and process conditions for obtaining carbon materials. Through the process of hydrothermal carbonization, the high carbon content of cellulosic materials can be converted into hydrothermal carbon materials without releasing CH_4_ and CO_2_ into the atmosphere. Hydrothermal carbonization, which takes place at lower temperatures, is a more energy-efficient process than pyrolysis at high temperatures of 600–1000 °C. Besides temperature, which is the main factor in the carbonization process, pH values can have a significant role in the structure and hydrothermal behavior of cellulose molecules. This consumption was investigated by Cui et al. by treatment of cotton fibers in subcritical water [26]. The authors found that pH has an influence on the morphology of the resulting spherical particles, carbon content, and calorific value. In order to improve the properties of the final activated carbon, the cotton was impregnated with urea [27] and then subjected to the processes of pyrolysis at 800 °C and hydrothermal carbonization. After activation with KOH, it was determined that there was an increase in pores and specific surface area, as well as that this material has a high affinity for iodine adsorption up to 2254.8 mg/g.

The concentrations of heavy metals in ground and surface waters are constantly increasing. The presence of most metals, some potentially harmful, in the environment is a consequence of human activity. There are different mechanisms of metals entering the water, washing streets, fields, unorganized landfills, coal and ore mining, and discharge of wastewater from various industrial plants [28,29]. If the measured concentrations of heavy metals are above the maximum allowed and desired limit, the toxic effects are manifested in a way that is harmful to people. The removal of metal ions from water, therefore, becomes inevitable to maintain the balance in the ecosystem. A very effective adsorbent for many pollutants is activated carbon, and much scientific research is directed toward obtaining carbon materials from various natural and waste sources [30,31,32,33,34,35,36]. To the best of our knowledge, the application of hydrothermally treated cotton, and specially cotton/polyester, for heavy metal removal in wastewater treatment has not been examined yet. Thus, in this study, hydrothermal carbonization and subsequent activation of waste cotton and cotton/polyester mix and characterization of obtained carbon materials as potential adsorbents of selected heavy metal ions were performed. Special attention is paid to the examination of the influence of further chemical activation on the porosity, specific surface area, the type and amount of surface oxygen groups, as well as the adsorption characteristics towards heavy metal ions of obtained materials.

## 2. Materials and Methods

### 2.1. Materials Preparation

Polysaccharide-based waste (cotton, C and mixed cotton/polyester ((50% cotton–50% polyester) yarn, C/P) generated at the factory SIMPO Dekor (Vranje, Serbia) was used as a precursor to obtain carbon materials. Hydrothermal carbonization of the yarn was carried out in a stainless steel autoclave with a Teflon insert at a temperature of 180 °C, under self-generated pressure, for 24 h. The reaction mixture consisted of 6 g of yarn, 40 cm^3^ of distilled water, and 0.015 g of citric acid as a catalyst. After hydrothermal carbonization, the solid product was filtered and washed with ethanol and distilled water. Two hydrothermally carbonized materials, C_HTC_ and C/P_HTC_, were obtained from cotton and mixed cotton/polyester yarn, respectively. Subsequent activation was performed with potassium hydroxide as an activating agent. Carbonized materials were mixed with KOH in the mass ratio 1:2 and heated in an electric furnace under a nitrogen atmosphere, up to 900 °C with a heating rate of 5 °C/min. The obtained samples were washed with distilled water until neutral reaction, dried at 100 °C overnight, and labeled C_aHTC_ and C/P_aHTC_.

### 2.2. Material Characterization

The morphological characteristics of the obtained materials were determined by scanning electron microscopy (Mira Tescan 3X, Tescan Orsay Holding, Brno, Czech Republic).

X-ray diffraction (XRD) patterns of all examined materials were recorded by Ultima IV Rigaku diffractometer, equipped with CuKα1,2 radiations. XRD spectra were recorded in the 2θ range of 10–60° (hydrothermally treated samples) and 15–60° (activated samples) in a continuous scan mode with a scanning step size of 0.02° and at a scan rate of 2° min^−1^.

Nitrogen adsorption and desorption isotherms were measured at the temperature of liquid nitrogen on a Micromeritics ASAP 2020 instrument (Micromeritics N.V./S.A., Brussels, Belgium) for all hydrothermally carbonized and activated samples. Using instrument ASAP 2020 software, the results of specific surface area S_BET_, external (S_ext_), and microporous (S_micro_) surface area, as well as total pore volume (V_total_) and volume of micropores (V_micro_), were obtained. Attenuated total reflectance Fourier transforms infrared spectroscopy (ATR-FTIR) was used to qualitatively analyze carbon materials’ surface oxygen groups. FTIR spectra were recorded in the 500–4000 cm^−1^ range, using ATR-FTIR Bomem MB-Series, Hartmann Braun (Quebeck, QC, Canada). For comparison, FTIR spectra of starting materials, cotton and cotton/polyester yarn, were also recorded. The amount of surface oxygen groups on examined carbon materials was determined by the acid-base titration method and this experiment was performed in triplicate for each material sample. For determination of the acidic and basic sites, small quantities (0.1 g) of each carbon material were mixed with 10 cm^3^ of 0.1 M NaOH and 0.1 M HCl, respectively, in 25 cm^3^ beakers. The beakers were sealed and shaken under a nitrogen atmosphere for 24 h. Afterward, filtered solutions were titrated with 0.05 M H_2_SO_4_ and 0.1 M NaOH to determine the amount of acidic and basic oxygen groups, respectively.

### 2.3. Adsorption Experiments

Adsorption of lead and cadmium ions on C_HTC_, C_aHTC_, C/P_HTC_, and C/P_aHTC_ was performed in a batch system by immersing 0.025 g of carbon adsorbent sample in 25 cm^3^ of adsorbate solution, initial concentration of 20 mg/dm^3^. The concentrations of Pb(II) and Cd(II) in the samples were determined using atomic absorption spectroscopy (AAS) at certain time intervals (5, 10, 15, 30, 60, 120 and 180 min). Experimental data were analyzed using pseudo-first (Equation (1)) [37] and pseudo-second-order (Equation (2)) [38] models for the optimization of the process and a better insight into the adsorption mechanism:

(1) $$q_{t}=q_{e}\times \left(1-e^{-k_{1}t}\right),$$

(2) $$q_{t}=q_{e}-{\left(\frac{1}{q_{e}}+k_{2}t\right)}^{-1},$$

where *q_e_* and *q_t_* (mg/g) are the adsorption capacity at equilibrium and at time *t* (min), *k*_1_ (1/min) and *k*_2_ (g/mg min) are the pseudo-first-order and pseudo-second-order rate constants.

Adsorption data obtained at different initial concentrations of lead ions (5, 15, 25, 35, 50, 75, and 100 mg/dm^3^) and cadmium ions (5, 7.5, 10, and 15 mg/dm^3^) were used to construct adsorption isotherms for all thermally modified samples. Langmuir [39] and Freundlich [40] isotherm models (Equations (3) and (4), respectively) were used to evaluate the affinity or capacity of the C_HTC_, C_aHTC_, C/P_HTC_, and C/P_aHTC_:

(3) $$q_{e}=\frac{Q_{0}K_{L}C_{e}}{1+K_{L}C_{e}}$$

(4) qe=Kf×Ce1n

where *C_e_* is the concentration of metal at equilibrium (mg/dm^3^), *Q*_0_ is the maximum adsorption capacity (mg/g), *K_L_* is a Langmuir adsorption equilibrium constant (dm^3^/mg) that is related to the apparent energy of sorption, *K_f_* (mg g^−1^(mg dm^−3^)^−1/*n*^) is Freundlich constant, and 1/*n* is the heterogeneity factor.

The possibility of reusing hydrothermally carbonized and subsequently activated waste yarn C_aHTC_ for the removal of Pb ions was investigated in a flow system. The material (0.03 g) was placed between two polyethylene frits in a cartridge. Under vacuum, a solution of Pb(II) ions (10 cm^3^ solution, initial concentration 5 mg/dm^3^) was passed through the cartridge at a flow rate of 1 cm^3^/min. After the adsorption cycle, the desorption of metal ions was performed by passing 5 cm^3^ of 2% HNO_3_ solution through the cartridge. The concentration of ions in the eluents (obtained after adsorption or desorption) was determined by atomic absorption spectroscopy. The adsorption/desorption procedure in the flow system was carried out in three cycles. The efficiency and capacity of adsorption and desorption processes (*E_ads_*, *q_a_*, *E_des_*, and *q_d_*, respectively) were calculated by Equations (5)–(8):

(5) $$E_{ads}=\frac{\left(c_{0}-c_{e}\right)}{c_{0}}\times 100,$$

(6) $$q_{a}=\frac{\left(c_{0}-c_{e}\right)}{m}\times 100,$$

(7) $$E_{des}=\frac{q_{d}}{q_{a}}\times 100,$$

(8) $$q_{d}=\frac{c_{d}V}{m},$$

where *c*_0_ (mg/dm^3^), *c_e_* (mg/dm^3^), and *c_d_* (μg/dm^3^) are the initial equilibrium and concentration of metal ions in the desorption solution, respectively, *V* (dm^3^) is the solution volume and *m* (g) is the adsorbent mass.

## 3. Results and Discussion

### 3.1. Material Characterization

**Figure 1:**
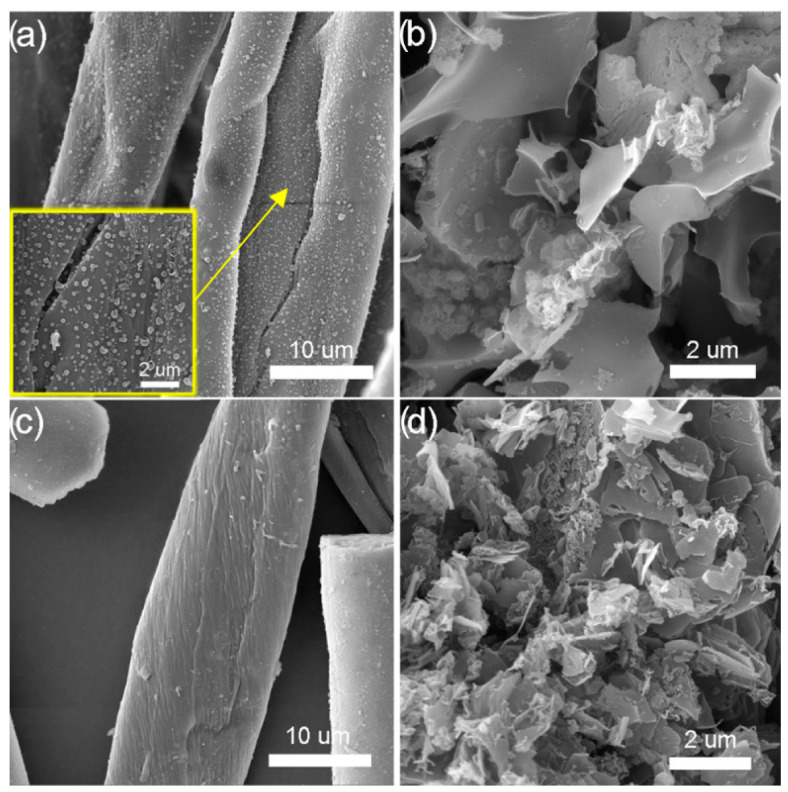
SEM photographs of samples: (**a**) C_HTC_, (**b**) C_aHTC_, (**c**) C/P_HTC_, and (**d**) C/P_aHTC_.

Scanning electron microscopy (SEM) was used to study the surface structure, topography, and morphology of hydrothermally modified and activated cotton and mixed cotton/polyester yarns (Figure 1).

Hydrothermally carbonized samples C_HTC_ and C/P_HTC_ (Figure 1a,c) retain the structure of the starting material: hydrothermally carbonized cotton and polyester components are characterized by spirally twisted fibers and smooth filament, respectively. In the case of sample C_HTC_, condensed smooth spherical particles, which are characteristic of hydrothermally carbonized materials, are observed on the surface. After the activation of the hydrothermally carbonized samples, with potassium hydroxide as an activating agent, the destruction of the fiber structure, both cotton and polyester components, in the samples is noticeable (Figure 1b,d).

**Figure 2:**
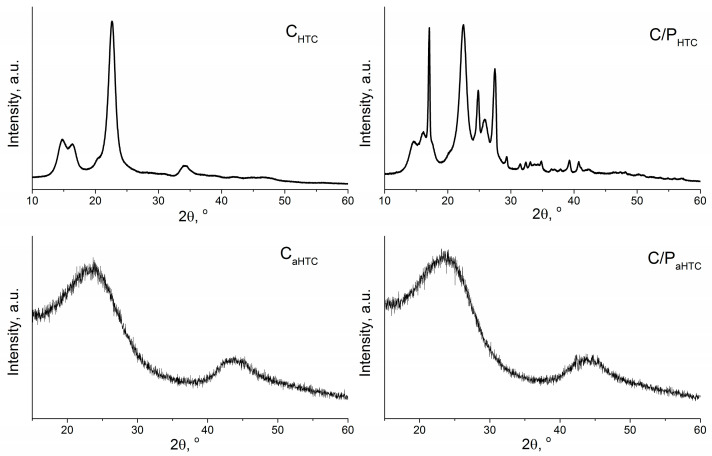
XRD spectra of hydrothermally treated and activated cotton and cotton/polyester yarn.

To examine the structure of hydrothermally treated and activated yarn XRD analysis was performed (Figure 2). XRD spectrum of C_HTC_ exhibits the typical diffraction peaks of the crystalline structure of cellulose I at 2θ = 14.7°, 16.4° and 22.6° [41], while XRD spectrum of C/P_HTC_ along with the peaks from cellulose, exhibits diffraction peaks characteristic for polyester at 2θ = 17.2°, 25.7° and 22.8° (superimposed with cellulose peak at 22.6°) [42].

As starting materials (cotton and cotton/polyester yarn) consist of pure cellulose and polyester, the XRD patterns of the hydrothermally treated samples, C_HTC_ and C/P_HTC_, confirm that these samples are not fully carbonized and that hydrothermal treatment leads to the partial decomposition of cellulose and its conversion to aromatic carbon network [43].

XRD spectra of chemically activated samples, C_aHTC_ and C/P_aHTC_, exhibit two typical broad peaks around 23.5° and 43.4° that indicate the disordered carbon structure and confirm that samples C_aHTC_ and C/P_aHTC_ are fully carbonized by applied activation.

**Figure 3:**
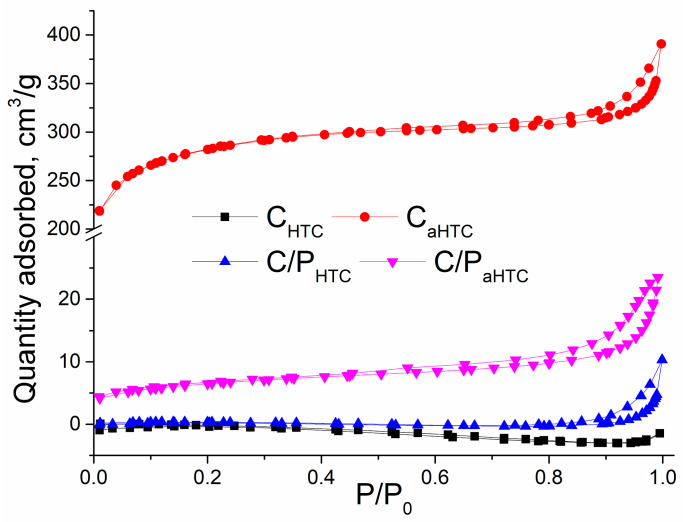
Adsorption–desorption isotherm of N_2_ on different adsorbents.

The observed changes in the morphology and structure of the activated samples can influence the specific surface area and affect the ability of the material to adsorb selected pollutants. Starting cotton and cotton/polyester yarns have no developed porosity, with the specific surface area immeasurably small and close to their geometric surface. Specific surface area and porosity of examined materials were determined from the adsorption-desorption isotherm of N_2_, shown in Figure 3.

The nitrogen adsorption-desorption curves for samples C_aHTC_, C/P_aHTC_, and C/P_HTC_ exhibit a hysteresis loop corresponding to type IV (C_aHTC_) and type V (C/P_aHTC_, C/P_HTC_) isotherms in the relative pressure range of 0.4–0.95, 0.5–0.95, and 0.8–0.95, respectively. This behavior is characteristic of mesoporous materials, which is in agreement with the average pore diameter (D_mean_) and the data presented in Table 1. The obtained results (Table 1) show that modification by hydrothermal carbonization does not lead to the development of the specific surface area of the material (S_BET_). A very low S_BET_ value for sample C_HTC_ makes it impossible to determine the pore volume and the mean pore diameter (D_mean_). The lack of porosity for sample C_HTC_ may be the consequence of the surface coverage by smooth condensed particles (Figure 1a). Sample C/P_HTC_ is a mesoporous material with an average pore diameter of 28.75 nm and a preserved fibrous structure of the starting precursor (Figure 1c). By applying the activation process, carbonized samples’ specific surface area and porosity increase while the average pore diameter decreases. After activation with KOH, there is a significant increase in the specific surface area for sample C_aHTC_ (872.2 m^2^/g). During activation, potassium is incorporated into the structure of the carbon material, forming various compounds that are washed after the process, leaving behind a free porous surface [44]. Based on mean pore diameter, samples C/P_HTC_ and C/P_aHTC_ can be classified as mesoporous, while sample C_aHTC_ shows a higher proportion of microporous surface area in the total specific surface area.

In addition to the specific surface and mean diameters of pores, the sorptive behavior of the material depends on the presence and type of surface oxygen groups. The quantitative composition of oxygenic acid and base groups present on the surface of carbonaceous materials was determined by the acid-base titration method (Table 1). Total acidic groups (carboxyl, lactone, phenol) are more abundant than basic ones for all samples of carbonaceous materials and are in the range of 3.763–4.866 mmol/g.

**Figure 4:**
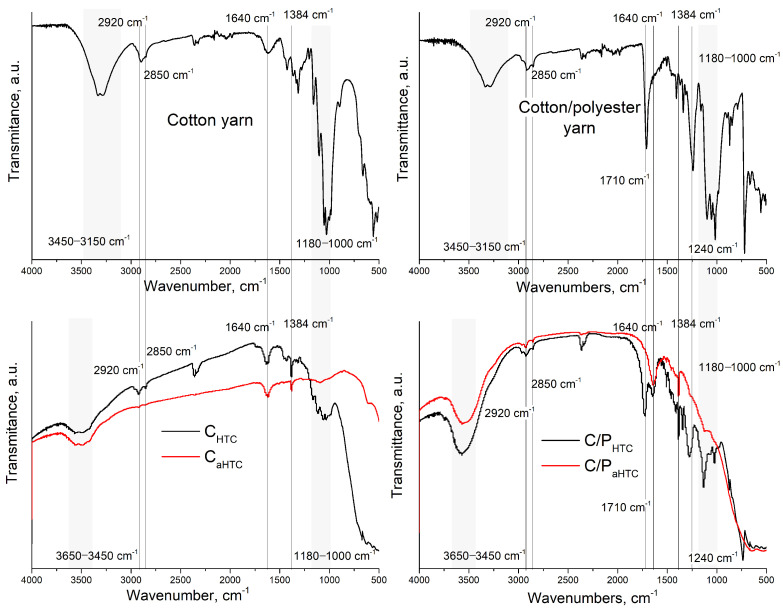
FTIR spectra of untreated cotton and mixed cotton/polyester yarns, and hydrothermally carbonized and activated samples.

Determination of functional groups and examination of changes occurring during the modification process was performed by recording FTIR spectra of precursors and modified samples. As seen in Figure 4, a broad band in the region of 3450–3150 cm^−1^ for precursors and 3650–3450 cm^−1^ for thermally treated samples may originate from the stretching vibrations of the O-H bond at carboxyl (COOH) or hydroxyl (OH) groups [45]. After activation, the increased temperature (900 °C) may lead to dehydration and loss of hydroxyl and carboxyl groups [46], which is visible from the decrease in the intensity of corresponding bands on FTIR spectra of activated samples. The peak at 2920 cm^−1^ is attributed to asymmetric, and at 2850 cm^−1^ to symmetric stretching vibrations of the C–H bond (carboxylic, methyl, or methylene groups) [47]. Also, a peak at 1640 cm^−1^ can be attributed to the bending vibrations of the O–H bond from carboxyl functional groups. The stretching vibration of the aliphatic C=C bond can also contribute to this peak. The deformational vibrations of the C–O bond in the carboxyl group can be observed as a peak at 1384 cm^−1^ [48].

On the FTIR spectra of the hydrothermally carbonized samples, C_HTC_ and C/P_HTC_, a significantly higher number of bands in the range 1800–1400 cm^−1^ is observed compared to the hydrothermally carbonized activated samples C_aHTC_, C/P_aHTC_. As the hydrothermal carbonization is performed under milder conditions and the precursors are not completely converted into carbon material, the FTIR spectra of these samples may also show some characteristic bands of the precursors but with the lower intensity compared with the FTIR spectra of cotton and mixed cotton/polyester yarns. Thus, the bands in the region 1180–1000 cm^−1^, observed in the samples C_HTC_ and C/P_HTC_, may originate from the stretching of the C-O bond (carboxyl groups, esters, ethers). The peaks at 1710 cm^−1^ and 1240 cm^−1^ indicate the presence of ester groups, and the peaks at 870 cm^−1^ and 720 cm^−1^ correspond to the bending vibrations of the C-H bond of the benzene ring in the polyester chain. After the activation of the C/P_HTC_ sample, the bands characteristic for the polyester component disappear (Figure 4).

### 3.2. Adsorption Experiments

**Figure 5:**
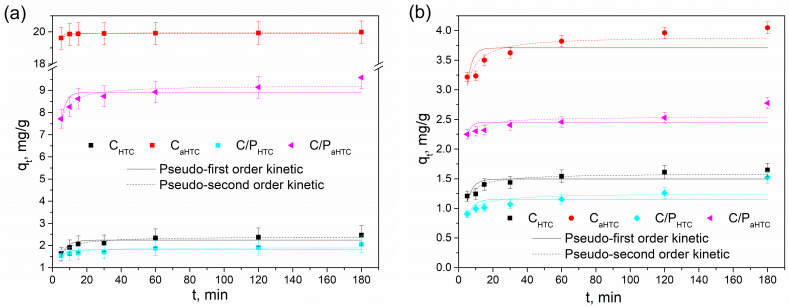
The influence of contact time on adsorption of (**a**) Pb and (**b**) Cd onto hydrothermally carbonized and activated cotton and mixed cotton/polyester.

Dependence of adsorption on time, as well as agreement of experimental data with pseudo-first- and pseudo-second-order models for the adsorption of (a) lead and (b) cadmium ions on thermally modified and activated cotton and cotton/polyester yarns (C_HTC_ and C_aHTC_, C/P_HTC_ and C/P_aHTC_) is shown in Figure 5. All examined samples showed higher capacities and rates of adsorption for the adsorption of lead ions, reaching the equilibrium after 30 min (Figure 5a). The highest adsorption capacity was observed for sample C_aHTC_, which also showed the fastest adsorption, reaching the removal efficiency of 99.9% after 15 min. Although the adsorption of cadmium ions onto examined samples can also be described as a fast process, examined samples showed much lower adsorption capacities for the removal of Cd(II) ions. These observations follow the kinetic parameters shown in Table 2. Based on correlation coefficients (*R*^2^) and comparison between experimentally obtained (*q_e_*_,*exp*_) and calculated (*q_e_*_,*mod*_) values of maximal adsorption capacities, adsorption of lead and cadmium ions onto examined samples can be described by the pseudo-second-order kinetic model, which indicates that heavy metal ions adsorption on carbonaceous materials takes place by a complex mechanism, and that chemisorption can play the most significant role in determining the overall reaction rate. The experimentally obtained values of the equilibrium adsorption capacities ranged from 2.06 to 19.98 mg/g for the adsorption of Pb(II) and from 1.52 to 4.05 mg/g for the adsorption of Cd(II) ions, following the sequence C_aHTC_ > C/P_aHTC_ > C_HTC_ > C/P_HTC_. Higher values of equilibrium adsorption capacities for Pb(II) ions may be a consequence of larger ionic radii and electronegativity compared to Cd(II) ions.

The highest values of equilibrium adsorption capacities obtained for the C_aHTC_ sample can be a result of the highest specific surface area (Table 1). However, a comparison between the values of specific surface area (Table 1) and the adsorption capacities of examined samples (Table 2) indicates that the specific surface area is not the key factor determining the adsorption capacities. The higher adsorption capacities of activated samples can also be influenced by the higher total amount of surface oxygen groups, which can serve as the main adsorption sites for metal ions. The achieved results are in agreement with literature data and scientific research [49,50,51,52,53].

**Figure 6:**
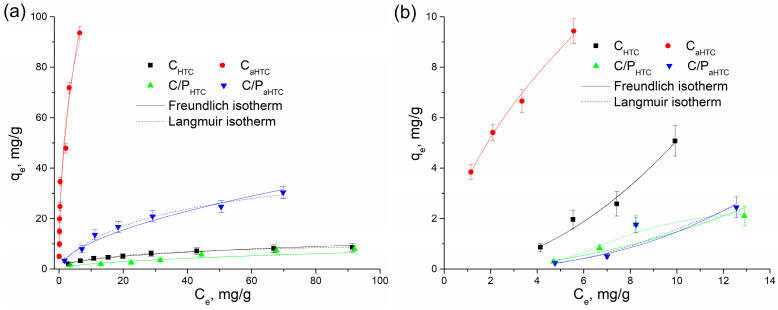
The influence of initial adsorbate concentration on adsorption of (**a**) Pb and (**b**) Cd onto hydrothermally carbonized and activated cotton and mixed cotton/polyester yarns.

To obtain additional information about the system, carbon material—lead and cadmium ions in an equilibrium state at a constant temperature, adsorption isotherms were used. The obtained equilibrium experimental results were fitted by Langmuir and Freundlich adsorption isotherms and shown in Figure 6, while the isotherm parameters are presented in Table 3. For all examined samples, adsorption capacities increase with the initial concentration of heavy metal ions. In the examined concentration range, the characteristic plot on the isotherm curve that indicates surface saturation was observed only for the adsorption of lead ions on C_HTC_ and C/P_HTC_ (Figure 6a). The increase in adsorption capacity is particularly pronounced for the C_aHTC_ sample during the removal of Pb(II) ions (Figure 6a). The maximum adsorption capacity (*q_max_*) obtained by the Langmuir model is very high, which can be attributed to the lack of equilibrium surface saturation in the examined concentration range and large specific surface area (Table 1). The process of adsorption on all thermally modified and activated yarns for Pb(II) ions, according to the obtained values of the correlation coefficient R^2^, is better described by the Langmuir model, which is most often the case during the removal of heavy metals from carbon materials whose precursors are cellulose materials [54,55]. A high value of the Freundlich constant indicates a high adsorption capacity, that is, a high affinity of the C_aHTC_ sample towards Pb(II) compared to other samples. Considering that the chemisorption process applies to Langmuir’s model, and the obtained values of 1/*n* < 1 indicate a heterogeneous adsorbent surface, it can be assumed that the adsorption mechanism is probably a combination of physical and chemical adsorption. In thermally modified cotton/polyester samples (C/P_HTC_, C/P_aHTC_), during the removal of Pb(II) ions, much lower values of adsorption capacity were obtained compared to thermally modified and activated cotton.

The adsorption process on C_HTC_ and C_aHTC_ for Cd(II) ions (Figure 6b) can be equally well described by both Langmuir and Freundlich isotherm models. The value of the heterogeneity factor less than 1 for the C_aHTC_ sample implies a relatively homogeneous surface, while adsorption occurs through the chemical process on the surface functional groups as active sites [56]. On the other hand, equilibrium adsorption data obtained for Cd(II) adsorption onto C/P_HTC_ and C/P_aHTC_ fits better with the Langmuir isotherm model, while the dimensionless heterogeneity factor 1/*n* has values approximately and greater than 1, indicating that with the increase in the concentration of Cd(II) ions, the free energy for further adsorption decreases [57]. Applied chemical activation improved the adsorption capacity of hydrothermally carbonized cotton, while in the case of cotton/polyester, activation did not bring the expected increase in adsorption capacity for the adsorption of Cd(II) ions.

Very few researchers have investigated the use of thermally modified cotton and cotton/polyester blends for metal ion adsorption. A comparison of results obtained in this study with the one found in the literature is given in Table 4.

According to Table 4, the adsorption capacities of hydrothermally treated and activated cotton and mixed cotton/polyester yarns investigated in this study are comparable or better than in cited studies. These results indicate that this way of modifying textile waste can produce an effective adsorbent for the removal of heavy metals, which corresponds to the principles of circular economy and sustainability.

### 3.3. Adsorption/Desorption

**Figure 7:**
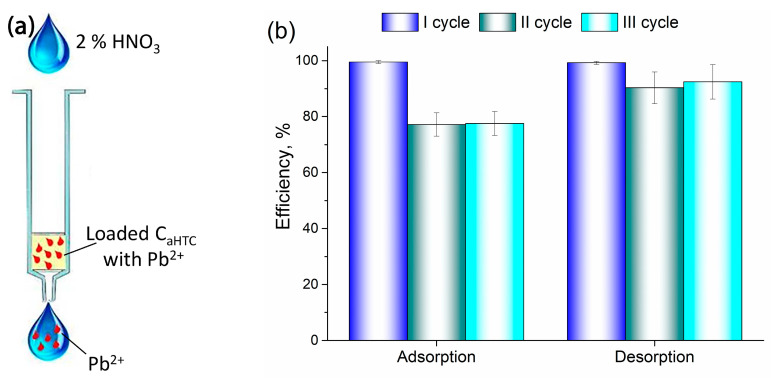
Scheme of lead ions desorption (**a**) and adsorption and desorption efficiency (%) of the C_aHTC_ sample (**b**).

The possibility of reusing thermally modified yarns was tested by the adsorption of lead ions on the C_aHTC_ sample, which showed the highest adsorption capacity. The adsorption/desorption procedure in the flow system was carried out in three cycles, where HNO_3_ was used as the desorption reagent (Figure 7a). The results of adsorption efficiency (%) and desorption efficiency (%) for all three cycles are shown in Figure 7b.

As can be seen from Figure 7b, the adsorption efficiency of Pb(II) ions in the first cycle on the C_aHTC_ sample is close to 100%, with a very high desorption efficiency of 99.23%. In the second and third cycles, lower adsorption efficiency values were obtained (77.16% and 77.53%), but desorption efficiencies were still relatively high (90.33% and 92.43%). The high efficiency of diluted nitric acid to desorb Pb(II) ions indicates the weak bound between adsorbate and adsorbent surface, leading to an assumption that Pb(II) ions are adsorbed through the mechanism of ion exchange or physisorption. Similar results on adsorption and desorption efficiency throughout several consecutive cycles were shown in the literature for the adsorption of Pb(II) ions onto carbonized cotton stalks [25] and adsorption of Pb(II), Cd(II), and Ni(II) ions onto carbonized cherry pits [35].

The results obtained from adsorption/desorption experiments have shown that hydrothermally carbonized and activated cotton yarn can be used as a highly efficient adsorbent for lead ions removal from water in at least three consecutive cycles of adsorption/desorption, enabling its practical application for wastewater treatment.

## 4. Conclusions

Following the increasing demand for cheap, available, and ecological adsorbents, waste materials with a large portion of natural cellulose polysaccharide were used in this work as starting raw materials to obtain effective carbon adsorbents for the removal of Pb(II) and Cd(II) ions from water. Hydrothermal carbonization was used to convert cotton and mixed cotton/polyester yarn to carbon adsorbents. Material characterization showed that additional chemical activation changed the morphology of materials and led to an increase in the specific surface area and the number of surface oxygen groups, consequently improving the affinity of obtained carbon materials for removing cations. Carbon adsorbent showing the highest values of Freundlich constant and Langmuir maximum adsorption capacity (3345.0 mg/g for Pb(II) ions adsorption) were obtained by hydrothermal carbonization and subsequent activation of cotton yarn. The adsorption of Pb(II) and Cd(II) ions onto hydrothermally carbonized and activated cotton and the mixed yarn is a relatively fast process following the pseudo-second-order kinetic, while the equilibrium adsorption process can be described by Langmuir isotherm model. The adsorption process most likely takes place by a complex mechanism, including physisorption and ion exchange on the surface oxygen groups. It was shown that hydrothermally carbonized and activated cotton yarns can be successfully reused in a flow system as a highly efficient adsorbent for lead ions in three consecutive adsorption/desorption cycles, with adsorption efficiency ranging from 100 to 77% throughout three cycles. The results of this research represent a significant contribution to solving the problem of removing heavy metal ions from water, using waste materials as adsorbents, and can serve as an example of the practical application of these materials in the purification of wastewater containing various heavy metals.

## Figures and Tables

**Table 1:** Surface properties of hydrothermally carbonized and activated cotton and mixed cotton/polyester yarns.

Sample	S_BET_, m^2^/g	S_ext_, m^2^/g	S_micro_, m^2^/g	V_total_, cm^3^/g	V_micro_, cm^3^/g	D_m_, nm	Amount of Surface Oxygen Groups, mmol/g		
							Basic	Acidic	Total
C_HTC_	0.003	-	-	-	-	-	0.983 ± 0.020	3.782 ± 0.017	4.765 ± 0.037
C/P_HTC_	0.64	0.122	0.516	0.01	4 × 10^−4^	28.75	0.345 ± 0.015	4.866 ± 0.012	5.211 ± 0.027
C_aHTC_	872.2	386.1	486	0.532	0.269	4.82	2.134 ± 0.008	3.940 ± 0.025	6.074 ± 0.033
C/P_aHTC_	21.75	19.03	2.72	0.035	0.002	8.93	2.333 ± 0.010	4.811 ± 0.015	7.144 ± 0.025

**Table 2:** Kinetic parameters for adsorption of Pb(II) and Cd(II) ions onto hydrothermally carbonized and activated cotton and mixed cotton/polyester yarns.

Metal Ion	Sample	Pseudo-First-Order Model			Pseudo-Second-Order Model			
		*R* ^2^	*k*_1_ (1/min)	*q_e,mod_* (mg/g)	*R* ^2^	*k*_2_ (g/(mg min))	*q_e,mod_* (mg/g)	*q_e,exp_* (mg/g)
Pb(II)	C_HTC_	0.71124	0.216	2.28	0.94000	0.159	2.42	2.46
	C/P_HTC_	0.28953	0.325	1.83	0.70164	0.325	1.93	2.06
	C_aHTC_	0.85576	0.842	19.91	0.91098	0.607	19.96	19.98
	C/P_aHTC_	0.54732	0.372	8.95	0.86901	0.100	9.26	9.59
Cd(II)	C_HTC_	0.47925	0.255	1.52	0.85098	0.295	1.61	1.65
	C/P_HTC_	0.23811	0.212	1.22	0.56231	0.243	1.31	1.52
	C_aHTC_	0.31121	0.337	3.75	0.78406	0.175	3.93	4.05
	C/P_aHTC_	0.10068	0.446	2.48	0.50107	0.417	2.57	2.78

**Table 3:** Langmuir and Freundlich parameters for adsorption of Pb(II) and Cd(II) ions onto hydrothermally carbonized and activated cotton and mixed cotton/polyester yarns.

Metal Ion	Sample	Langmuir Isotherm			Freundlich Isotherm		
		*q_max_* (mg/g)	*b* (dm^3^/mg)	*R* ^2^	*K_f_* ((mg/g)/(mg/dm_3_)^1/n^)	1/n	*R* ^2^
Pb(II)	C_HTC_	260.8	0.006	0.99999	1.683	0.372	0.98237
	C/P_HTC_	81.6	0.004	0.94043	0.361	0.694	0.92571
	C_aHTC_	3345.0	0.012	0.99999	40.596	0.448	0.95892
	C/P_aHTC_	49.7	0.046	0.98189	3.634	0.501	0.97498
Cd(II)	C_HTC_	1357.0	4.62 × 10^−5^	0.93807	0.063	1.906	0.96911
	C/P_HTC_	2.2	1.01 × 10^−6^	0.92372	0.086	1.275	0.71281
	C_aHTC_	843.4	0.00414	0.97853	3.480	0.573	0.98945
	C/P_aHTC_	2.4	4.81 × 10^−13^	0.94990	0.026	1.807	0.76007

**Table 4:** Comparison of maximal adsorption capacities of carbon adsorbents based on cotton and cotton/polyester for removal of heavy metal.

Material	Modification	Initial Concentration, mg/dm^3^	Heavy Metal	Adsorption Capacity *q_max_*, mg/g	Reference
Cotton fabric	Pyrolysis + Activation H_3_PO_4_	80–500	Pb(II)	361.54	[20]
Cotton/polyester fabric 75:25		80–500		385.77	
Cotton fibre	Microwave-assisted carbonization	0.05–0.4	Hg(II)	169.2	[58]
Cotton stalk	Pyrolysis	100–500	Pb(II)	146.78	[25]
Cotton stalk	Hydrothermal carbonization + Activation KOH	5–300	Cd(II)	30.40	[17]
Cotton stalk	Pyrolysis	10–80	Pb(II)	42.55	[59]
		0.1–1.0	Cd(II)	0.53	
		1.0–10	Ni(II)	5.25	
		0.1–1.0	Co(II)	0.54	
	Pyrolysis + H_2_SO_4_	10–80	Pb(II)	38.76	
		0.1–1.0	Cd(II)	0.53	
		1.0–10	Ni(II)	2.21	
		0.1–1.0	Co(II)	0.52	
	Pyrolysis + NaOH	10–80	Pb(II)	37.59	
		0.1–1.0	Cd(II)	0.51	
		1.0–10	Ni(II)	2.05	
		0.1–1.0	Co(II)	0.50	
	Pyrolysis + H_2_C_2_O_4_	10–80	Pb(II)	44.64	
		0.1–1.0	Cd(II)	0.65	
		1.0–10	Ni(II)	6.20	
		0.1–1.0	Co(II)	0.52	
Waste cotton yarns	Hydrothermal carbonization	5–100	Pb(II)	260.8	This study
Waste cotton/polyester yarns		5–100		81.6	
Waste cotton yarns		5–15	Cd(II)	3345.0	
Waste cotton/polyester yarns		5–15		49.7	
Waste cotton yarns	Hydrothermal carbonization + Activation KOH	5–100	Pb(II)	1357.0	
Waste cotton/polyester yarns 50:50		5–100		2.2	
Waste cotton yarns		5–15	Cd(II)	843.4	
Waste cotton/polyester yarns 50:50		5–15		2.4	

## Data Availability

The original contributions presented in the study are included in the article, further inquiries can be directed to the corresponding author.
